# Multiple sexual selection pressures drive the rapid evolution of complex morphology in a male secondary genital structure

**DOI:** 10.1002/ece3.1721

**Published:** 2015-09-23

**Authors:** Stephen R. Frazee, John P. Masly

**Affiliations:** ^1^ Department of BiologyUniversity of OklahomaNormanOklahoma73019

**Keywords:** Competitive fertilization, mating success, morphological evolution, secondary genitalia

## Abstract

The genitalia of internally fertilizing taxa represent a striking example of rapid morphological evolution. Although sexual selection can shape variation in genital morphology, it has been difficult to test whether multiple sexual selection pressures combine to drive the rapid evolution of individual genital structures. Here, we test the hypothesis that both pre‐ and postcopulatory sexual selection can act in concert to shape complex structural variation in secondary genital morphology. We genetically modified the size and shape of the posterior lobes of *Drosophila melanogaster* males and tested the consequences of morphological variation on several reproductive measures. We found that the posterior lobes are necessary for genital coupling and that they are also the targets of multiple postcopulatory processes that shape quantitative variation in morphology, even though these structures make no direct contact with the external female genitalia or internal reproductive organs during mating. We also found that males with smaller and less structurally complex posterior lobes suffer substantial fitness costs in competitive fertilization experiments. Our results show that sexual selection mechanisms can combine to shape the morphology of a single genital structure and that the posterior lobes of *D. melanogaster* are the targets of multiple postcopulatory selection pressures.

Genitalia are generally regarded as one of the most rapidly evolving traits because of the remarkable diversity of structures and morphologies that are observed among even the youngest pairs of species (Eberhard 1985; Hosken and Stockley 2004; Simmons 2014). One of most striking features of this diversity is the structural complexity of male external morphological traits. In arthropods, male genitalia are often comprised of a complex array of structures including those that insert directly into the female's gonopore (primary intromittent genitalia), structures that insert into the female, but not directly into the gonopore (secondary intromittent genitalia), and structures that come into contact with the female, but remain external during mating (secondary nonintromittent genitalia). Primary intromittent structures are functionally important for insemination and are closely tied with fertilization success, whereas secondary genital structures (both intromittent and nonintromittent) are thought to function primarily in grasping the female to secure a tight genital coupling (Eberhard 1985).

The evolutionary consequences of variation in primary male genital structures have been deduced in several taxa by studying intraspecific natural variation in morphology. The results of these experiments are consistent with postcopulatory sexual selection as the predominant force that appears to drive the evolution of primary genital morphology (e.g., Arnqvist et al. 1997; Córdoba‐Aguilar et al. 2003; House and Simmons 2003; Stockley et al. 2013; Simmons 2014; Simmons and Firman 2014). Recent advances in technology have also made it possible to directly test the reproductive consequences of variation in genital morphology. In particular, laser microsurgery provides an effective technique for modifying the size and/or shape of individual genital structures (or ablating them altogether) without causing collateral damage to nearby structures that might also be important for mating. For example, in the seed beetle *Callosobruchus maculatus*, laser microsurgery was used to shorten the length of sclerotized cuticular spines present on the male primary intromittent genitalia. Males with surgically shortened spines suffered lower fertilization success and transferred a smaller volume of ejaculate to the female's hemolymph through genital wounds compared to males with longer spines (Hotzy et al. 2012). This result confirms that postcopulatory sexual selection drives the evolution of larger spine length in this species.

Laser microsurgery has also been used to study the reproductive consequences of variation in secondary intromittent genital structures. *Drosophila ananassae* males possess a pair of hook‐like genital spines that insert into the female external genitalia (although not directly into the gonopore) during mating. Males with surgically shortened spines suffer a reduced ability to achieve copulation when paired individually with a female. This difficulty to achieve and maintain genital coupling was even more pronounced in a social environment where males competed with one another for mates (Grieshop and Polak 2012). *D. bipectinata* males possess genital spines similar to those of *D. ananassae*, and quantitative reductions in genital spine length also result in reduced copulation success in both individual and socially competitive environments (Polak and Rashed 2010). These results demonstrate the functional importance of genital spines for coupling male and female genitalia together during mating, and show that these secondary genital structures have been the target of precopulatory sexual selection. Interestingly, in both *D. ananassae* and *D. bipectinata*, males with shortened spines sometimes do manage to achieve genital coupling when paired individually with a female. In these cases, insemination success and fertilization rates do not significantly differ compared to controls (Polak and Rashed 2010; Grieshop and Polak 2014), which suggests that postcopulatory sexual selection does not contribute to the evolution of spine length in these two species.

Despite great progress in understanding the reproductive consequences of morphological variation in male genitalia, the relationship between pre‐ and postcopulatory selection, their relative strengths, and the type of genital structures they typically target still remain largely unknown. A comparative study of intromittent and nonintromittent genital morphology and complexity among *Gerris* water striders identified two patterns that suggest pre‐ and postcopulatory selection act on different types of structures and with different strengths (Rowe and Arnqvist 2012). First, genital structures show correlated evolution with different proxies for sexual selection in this genus. Male intromittent genitalia are positively correlated with male internal reproductive organ size, indicative of postcopulatory sexual selection, whereas nonintromittent genitalia are positively correlated with sexual dimorphism in body shape, indicative of precopulatory sexual selection. Second, intromittent genital structures show greater morphological divergence and complexity than nonintromittent structures, and divergence between intromittent and nonintromittent structures is uncorrelated. Together, these results suggest that in this genus, postcopulatory selection shapes intromittent genital morphology, precopulatory selection shapes nonintromittent genital morphology, and postcopulatory sexual selection is stronger than precopulatory sexual selection in shaping complex genital morphologies.

Although secondary genitalia often show reduced morphological complexity and diversity relative to primary genitalia, there are examples of male genital grasping devices that display morphological divergence among species similar to that typically observed in primary structures. One example of this occurs among *Glossina* tsetse flies that possess dramatic differences in cerci morphology among species. These structures grasp the outside of the female's abdomen during mating, and variation in their morphology can affect female sperm storage, ovulation, and remating behavior, consistent with a history of postcopulatory sexual selection (Briceño and Eberhard 2009a,b). In addition to the morphological differences among species, male copulation behavior also appears to be a target of postcopulatory sexual selection, as males move their intromittent genitalia differently during mating to stimulate the female (Briceño et al. 2015).

Some species of *Drosophila* also possess intromittent secondary genital grasping devices that display striking divergence in morphology among sister species. The posterior lobes of the male genital arch are newly evolved, bilaterally symmetrical sclerotized cuticular projections that are found only among the four species of the *D. melanogaster* species complex (*D. melanogaster*,* D. simulans*,* D. mauritiana*, and *D. sechellia*; Jagadeeshan and Singh 2006). During copulation, the posterior lobes insert between female abdominal segments VII and VIII, but make no direct contact with the female's primary genitalia or internal reproductive organs (Kamimura 2010). The posterior lobes show dramatic differences in both size and shape among these species (see Ashburner et al. 2005; fig. 33.1), and despite a long‐standing interest and effort to understand the importance of these structures during mating and the evolutionary forces that shape variation in their morphology, direct tests have been difficult.

Recently, a laser microsurgery approach was used to modify the size and shape of the posterior lobes of *D. simulans* to test the importance of morphology on mating, fertilization success, and reproductive isolation between species (LeVasseur‐Viens et al. 2015). In particular, three specific modifications of the posterior lobe were generated to alter lobe morphology, and males that possessed altered posterior lobes were allowed to mate with *D. simulans* females to measure the effect of morphological variation on pre‐ and postcopulatory reproductive phenotypes. The results of these experiments showed that the posterior lobes are necessary for copulation and that males with altered lobe morphologies suffered reduced copulation success compared to males with unaltered lobes in a socially competitive mating environment. However, there was no effect of posterior lobe alteration on copulation duration or sperm transfer, and females were just as likely to fertilize eggs from an altered male as they were from an unaltered male. These results suggest that posterior lobe morphology does not affect postcopulatory reproductive success and that precopulatory sexual selection is the predominant force that drives the evolution of complex posterior lobe morphologies.

Here, we use a nonsurgical approach to modify posterior lobe size and shape in *D. melanogaster* to test the potential importance of postcopulatory sexual selection in shaping male genital morphology. Our experiments take advantage of the GAL4‐UAS binary inducible gene expression/repression system (reviewed in Duffy 2002), which allows us to genetically abrogate posterior lobe development to generate variation in different aspects of morphology without negative pleiotropic consequences on other male reproductive phenotypes. We test a large collection of individuals that possess variation in posterior lobe size and shape to determine the importance of quantitative variation in morphology on several pre‐ and postcopulatory reproductive measures and identify the aspects of morphology that affect fitness. In contrast to the findings in *D. simulans*, our results show that both pre‐ and postcopulatory sexual selection shape complex posterior lobe morphology in *D. melanogaster* and that multiple mechanisms of postcopulatory sexual selection contribute to the evolution of morphology.

## Materials and Methods

### *Drosophila* stocks

Fly stocks were maintained and crossed on cornmeal–yeast–molasses medium at room temperature (23–25°C) on a 12‐h light:dark cycle. All stocks were maintained at similar densities for several generations prior to performing all mating experiments. We used the following *D. melanogaster* stocks to construct the genotypes used in our experiments:

*w* ^1118^
Canton S
*w, P[UAS‐Dcr‐2.D]1*
*y v;; P[TRiP.UAS‐PoxnIR.JF02136]attP2*
*y w; Pin/CyO; P[mcd8::GFP]*
*y w; apGAL4, UAS‐GFP/CyO*
*P[elav‐GAL80.Sb]/CyO; MKRS/TM6B Tb*

For all experiments we performed, we collected virgin females and males using light carbon dioxide anesthesia and aged all individuals for 3–4 days at room temperature. Males and females were then aspirated into mating chambers or food vials by mouth. Because some mutant alleles of *yellow* (*y*) are known to affect aspects of male courtship behavior (Drapeau et al. 2003), all genotypes used to assess mating and courtship behavior were constructed to exclude the *y* allele.

### Genetic modification of posterior lobe morphology

To abrogate posterior lobe development, we silenced the expression of *Pox neuro* (*Poxn*), a gene important for development of the external genitalia in *D. melanogaster* (Boll and Noll 2002), using RNA interference (RNAi). Because *Poxn* is expressed in both the developing male genitalia and in parts the central and peripheral nervous systems (Boll and Noll 2002), we sought to limit the domain in which we silenced *Poxn* expression to target the cells that give rise to the posterior lobe. To do this, we used an *enhancer‐GAL4* driver that has partially overlapping expression with *Poxn* during development. Specifically, we took advantage of an *apterous‐GAL4* (*apGAL4*) transgene, which overlaps *Poxn* expression in the primordial posterior lobe cells, to drive expression of a *UAS* transgene that encodes a *Poxn* inverted repeat sequence (*UAS‐PoxnIR*). To increase the efficacy of RNAi gene silencing, we also overexpressed *Dicer‐2* (*UAS‐Dcr‐2*) in the *apGAL4; UAS‐PoxnIR* genotype (hereafter, *“Dcr2; ap>PoxnIR*”). Prior to performing crosses to quantify mating and courtship, we outcrossed each of the transgenic stocks described above to Canton S to minimize genetic background effects that might confound our interpretation of variation in posterior lobe morphology on reproductive phenotypes (*e.g*., seminal fluid protein variation, behavioral variation).

### Mating assays and courtship behavior

Because *Poxn* is expressed in the adult nervous system where it functions in specifying aspects of male courtship behavior (Boll and Noll 2002), we silenced the activity of the GAL4 protein in the nervous system prior to mating assays using an *embryonic lethal abnormal vision‐GAL80* (*elavGAL80*) transgene. This transgene produces GAL80 protein in all neural tissues (Mosca et al. 2012); GAL80 binds to the transcriptional activation domain of GAL4 and represses its ability to activate genes downstream of the *UAS* binding site (reviewed in Melcher 1997). To ensure that apGAL4 was effectively silenced by elavGAL80 in the nervous system, we dissected the brain and ventral nerve cord from *UAS‐Dcr‐2; apGAL4, UAS‐GFP/+; UAS‐PoxnIR/elavGAL80* adult males immediately after their mating trial. Only data from those males that showed no detectable GFP expression in neural tissue were used in our analyses. We also confirmed the ability of elavGAL80 to silence apGAL4 at other developmental stages by examining the nervous system of stage 11–17 embryos.

To quantify mating and courtship behaviors, we paired a single male and a single white‐eyed (*w*) female in a 2‐cm‐diameter x 1‐cm‐deep cylindrical mating chamber and video‐recorded their interactions for 10 min or until copulation was achieved. We analyzed the recordings using a custom coding scheme in Noldus The Observer^®^ XT software and quantified Courtship Index (CI) and Wing Extension Index (WEI) as a measure of a male's ability to successfully perform proper courtship behavior. CI was calculated as the proportion of time a male spent performing any stereotypical *Drosophila* courtship behavior, and WEI was calculated as the proportion of time a male spent performing wing extension during courtship (Siegel and Hall 1979). We performed mating experiments and quantified courtship for a minimum of *n* = 10 males for each genotype we studied.

### Quantification of reproductive measures

Males and females used to quantify pre‐ and postcopulatory reproductive measures were paired within an hour of first daylight in eight‐dram food vials. To mimic the mating chamber dimensions described above, the cotton ball of each vial was pushed down toward the surface of the food to generate an arena of similar dimensions. Each pair was observed for a period of up to five hours or until copulation ceased. For each successfully copulating pair, we recorded copulation duration and separated males and females immediately after copulation ended. Females were then frozen within one hour to quantify male sperm transfer. We dissected the female reproductive tract in a drop of 1X PBS and separated the spermathecae, seminal receptacle, and uterus/common oviduct. The contents of these organs were removed and spread on a glass slide, allowed to dry, fixed in 3:1 methanol:acetic acid, and stained with 0.2 *μ*g/mL DAPI to visualize and quantify the number of sperm nuclei.

Individual males were isolated for 3 days after their initial mating to replenish expended sperm before being mated with new *w* virgin females. Adults were transferred to new food vials every 3 days for 15 days. We recorded the number of eggs that were laid, number of eggs that hatched, and the total number of offspring that emerged from each of the five vials. Offspring were scored up to day 19 after the adults were first introduced into each new vial. We tested a minimum of *n* = 30 males of each genotype for these mating assays, and each set of mating experiments was scored blind with respect to male genotype.

### Competitive fertilization assays

We mated single *w* females with a single male from either a focal genotype or a reference genotype and then mated them with a male from the reciprocal genotype. Males from the reference genotype were heterozygous for the dominant visible markers *Pin* and *Curly* to allow us to easily assign paternity in each of the crosses. For the first cross in the sequence, we visually confirmed that mating had occurred before removing the males and allowing the females to remain in their mating vials for 24 h. We then paired females with the second male. These pairs were allowed to remain together overnight, as females are often slow to re‐mate after their initial mating (reviewed in Singh et al. 2002). We removed the second male from the vial and transferred the female to a new food vial 1 day afterward and then every 3 days for a total of 24 days after the initial mating had occurred. We scored the number of offspring produced from each of the eight vials and calculated the proportion of progeny sired by the focal male when they mated first (P_1_) and when they mated second (P_2_). Only data from those females that produced at least one offspring from the second mating were included in our analyses.

### Posterior lobe morphological measurements

**Figure 1:**
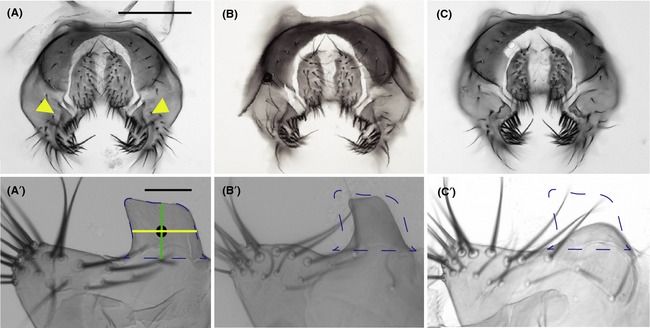
Genetic manipulation produces variation in *Drosophila melanogaster* posterior lobe morphology. (A) Whole‐mount dissection of wild‐type (Canton S) external male genitalia of *D. melanogaster*. Yellow arrowheads point to the posterior lobes projecting out from the epandrium. (B) External genitalia of intermediate‐lobed *ap>PoxnIR* males. (C) External genitalia of nub‐lobed *Dcr2; ap>PoxnIR* males. The structures of the hypandrium (not pictured) for all genotypes were visually inspected for defects and none were observed. Scale bar is 100 *μ*m. (A’–C’) Posterior lobes from each respective genotype shown in panels (A–C). The dashed blue overlay shows the outline of a typical wild‐type posterior lobe. The area within this outline was used to represent posterior lobe size. Posterior lobe length (green line) and width (yellow line) were measured through the centroid (black circle) of the outline. Scale bar is 25 *μ*m.

Whole external genitalia shown in Figure 1 were dissected from adult males and incubated in 10% NaOH at 80°C for 30 min to remove soft tissues. The cleared cuticular structures were rinsed in 1X PBS, mounted in 80% glycerol, and imaged at 100× magnification using bright field microscopy.

Left and right posterior lobes and lateral plates were dissected, mounted in polyvinyl alcohol on glass slides under coverslips, and imaged at 200× magnification. The outline of each posterior lobe was manually traced using ImageJ (Rasband 1997‐2014) and enclosed with an artificial baseline drawn in line with the lateral plate. Each closed contour was then converted into (*x*,* y*) coordinates that were used to represent posterior lobe morphology. We performed elliptical Fourier analysis (EFA) using a previously published algorithm (Kuhl and Giardina 1982; Ferson et al. 1985) and used 20 harmonic iterations of the Fourier series to generate 80 elliptical Fourier coefficients for each posterior lobe we analyzed. We analyzed the EFA output using principal components analysis (PCA) to reduce the number of variables that describe variation in posterior lobe morphology. Elliptical Fourier coefficients were adjusted to standardize location, orientation, and handedness within the coordinate plane prior to PCA. We selected one posterior lobe at random from each individual we dissected to include in our PCA, and PCA was performed using singular value decomposition of the elliptical Fourier coefficient data matrix. Left and right foreleg tibias were dissected and measured to provide an estimate of overall body size for each male we tested in our study. Tests were performed on both the raw measurements and measurements that had been corrected for differences body size and produced similar results.

Although EFA represents complex morphologies that lack reliable landmarks with high precision (Kuhl and Giardina 1982; Lestrel 1997), the exact morphological correlates of EFA output are often difficult to assign. Therefore, as a complement to EFA, we made three additional posterior lobe measurements. We measured the area of each posterior lobe as the area enclosed within the lobe outline, and the length and width of the posterior lobe as drawn through the centroid of the lobe outline (see Fig. 1A′; McNeil et al. 2011).

### Statistical analyses

All proportion data obtained for CI and WEI were transformed using arcsine (square root(proportion)) before performing analysis of variance and Tukey's post hoc tests. The effect of variation in posterior lobe morphology on pre‐ and postcopulatory reproductive measures was tested using multivariate analysis of variance (MANOVA) with the reproductive measures as the response variables and the representations of posterior lobe morphology plus tibia length as explanatory variables. We performed subsequent univariate *F* tests to identify the effect of particular aspects of morphology on each reproductive measure.

Paternity data from competitive fertilization assays are binomially distributed (House and Simmons 2006; Firman and Simmons 2008) and were thus analyzed using a generalized linear model. In these models, the numerator of the response variable was the number of offspring sired by the focal male and the denominator was the total number of offspring produced by the female. The explanatory variable was the direction of the cross with respect to male genotype. Because these data were overdispersed, we corrected for overdispersion by fitting the model using quasibinomial distributed errors with a logit link function, and tested the fit of the data to the model using an *F* statistic instead of a chi‐square statistic.

We preformed all statistical analyses using R release 3.1 (R Core Team 2014). Figures were constructed using the R package ggplot2 (Wickham 2009).

## Results

### The posterior lobes are necessary for successful mating

To abrogate posterior lobe development, we used a GAL4‐UAS approach to silence the expression of *Poxn*, a gene important for development of the external genitalia in *D. melanogaster* (Boll and Noll 2002), in the cells that give rise to the posterior lobe using RNAi. This allowed us to almost completely ablate the posterior lobe without affecting the morphology of other external genital structures that are used during mating (e.g., claspers, sensory bristles; Fig. 1). Because *Poxn* and the GAL4 driver we used (*apGAL4*) are both expressed in parts of the nervous system during some developmental stages (Bopp et al. 1989; Cohen et al. 1992; Lundgren et al. 1995; Boll and Noll 2002), the possibility exists that any overlap between the *Poxn* and *ap* neural circuits could affect male mating behaviors. To remove these potentially confounding effects of abnormal mating behavior, we inhibited GAL4 activity in the neuronal tissues of *Dcr2; ap>PoxnIR* males using a pan‐neuronal *elavGAL80* transgene (Mosca et al. 2012). The genotypes we constructed to abrogate posterior lobe development produced two distinct posterior lobe phenotypes: *Dcr2; ap>PoxnIR* males that possess a “nub‐lobed” phenotype compared to control males (Fig. 1A′ vs. 1C′) and *ap>PoxnIR* males that possess an “intermediate‐lobed” phenotype compared to control males (Fig. 1A′ vs. 1B′).

We tested the ability of males to mate successfully by pairing them individually with a single *D. melanogaster w* female and observed their interactions for a 10‐min time period. We found no significant differences in CI (*F*_6,64_ = 0.94, *P *=* *0.47) or WEI (*F*_6,64_ = 1.76, *P *=* *0.12) among the seven genotypes we tested (Fig. S1). This result shows that our genetic manipulations have no deleterious effects on male courtship behaviors. Our results also show that control males exhibit normal copulation behaviors and achieve copulation with an appreciable frequency during a 10‐min observation period (24 of 40 trails; Movie S1). This was true even for intermediate‐lobed *ap>PoxnIR* males compared to control males (4 of 10 trails; Fig. 1B′). In contrast, although nub‐lobed *Dcr2; ap>PoxnIR/elavGAL80* males display normal courtship and attempted mounting behavior, they fail to secure prolonged genital contact with the female despite repeated attempts and never achieve copulation (0 of 11 trails; Movie S2).

As an additional test of the importance of the posterior lobe for copulation, we set up matings in food vials and recorded male–female interactions during a five‐hour observation period. Consistent with the mating chamber results, nub‐lobed *Dcr2; ap>PoxnIR* males make repeated attempts to copulate, but never achieved copulation during the extended observation period. Moreover, although both control and intermediate‐lobed *ap>PoxnIR* males sired abundant progeny (mean ± 1 SEM: 104 ± 1.0 and 84 ± 5.0, respectively), nub‐lobed *Dcr2; ap>PoxnIR* males sired none. The results of our mating experiments thus show that the posterior lobes in *D. melanogaster* are necessary for males to secure genital coupling and copulate, consistent with the results from laser microsurgery experiments in this species (LeVasseur‐Viens et al. 2015). The high‐speed video recordings also suggest that the posterior lobes appear to serve as a “hook‐like” device as previously hypothesized (Jagadeeshan and Singh 2006), and may also serve as a guide for the male genitalia during genital coupling (S. R. Frazee, pers. obs.).

### Variation in posterior lobe morphology affects several reproductive measures

The genotypes we constructed to abrogate posterior lobe development yield two interesting observations. First, we were unable to completely ablate the posterior lobe in the *Dcr2; ap>PoxnIR* genotype, yet these males were still unable to achieve copulation using their nub‐like structures (Fig. 1C′). Thus, there appears to be a minimum requirement for posterior lobe size/shape that allows males to secure genital coupling, similar to what is observed for secondary, intromittent genitalia in other *Drosophila* species (Polak and Rashed 2010; Grieshop and Polak 2012; LeVasseur‐Viens et al. 2015). Second, variation in *Poxn* expression level can give rise to variation in posterior lobe morphology (Fig. 1A′–1C′). In particular, *ap>PoxnIR* males that experience less severe *Poxn* silencing during posterior lobe development possess reduced lobe morphologies compared to controls (Fig. 1A′ vs. 1B′). However, their posterior lobes are morphologically sufficient to enable these males to secure genital coupling and copulate with the female. This presents an opportunity to test the effect(s) of quantitative variation in posterior lobe morphology on male and female reproductive measures.

We mated *ap>PoxnIR* males, and males from four control genotypes (Canton S, plus *apGAL4*,* PoxnIR*,* UAS‐Dcr2; apGAL4* to control for possible effects of these transgene insertions on reproductive phenotypes), with individual *w* females in food vials and quantified several reproductive measures. Briefly, we recorded copulation duration and sperm transfer from a single mating, before using the same male in a second mating to record female oviposition, egg hatch, and the total number of offspring that emerged from each of 5 vials over a 15‐day period. This design allowed us to test the effect of posterior lobe morphology from the same male on both pre‐ and postcopulatory reproductive measures.

To understand the particular aspects of posterior lobe morphology that might affect reproductive success, we measured morphology using two standard approaches for *D. melanogaster* (Liu et al. 1996; McNeil et al. 2011). First, we performed EFA followed by PCA to represent variation in posterior lobe morphology among the five genotypes we tested in this set of mating experiments. In this dataset, the first 3 principal components (PC1–PC3) explain roughly 80 percent of the variation in morphology. Second, we measured the area of each posterior lobe as the area enclosed within the lobe outline, and the length and width of the posterior lobe as drawn through the centroid of the lobe outline (Fig. 1A′). These measures correlate well with PC1–PC3 for *D. melanogaster* posterior lobe morphology. In particular, PC1 primarily captures posterior lobe area (*r* ^2^ = 0.83), PC2 captures length‐to‐width ratio (L:W; *r* ^2^ = 0.36), and PC3 captures posterior lobe length (*r* ^2^ = 0.26).

**Figure 2:**
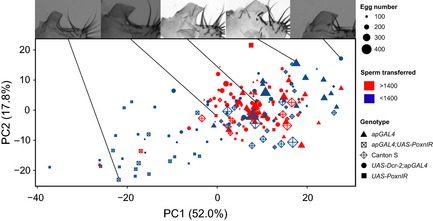
Variation in posterior lobe morphology affects reproductive measures. Variation in posterior lobe morphology is shown across the distribution of principal components 1 and 2 (PC1 and PC2). The number of eggs oviposited by females after mating with a male of a particular genotype is shown by the size of each plotted point. Sperm transfer amount is binned into two classes based on the median value for the entire sample. Images of posterior lobes show representative examples of the distribution in morphology across the PC1–PC2 axes. Numbers in parentheses show the proportion of morphological variation explain by PC1 and PC2.

**Figure 3:**
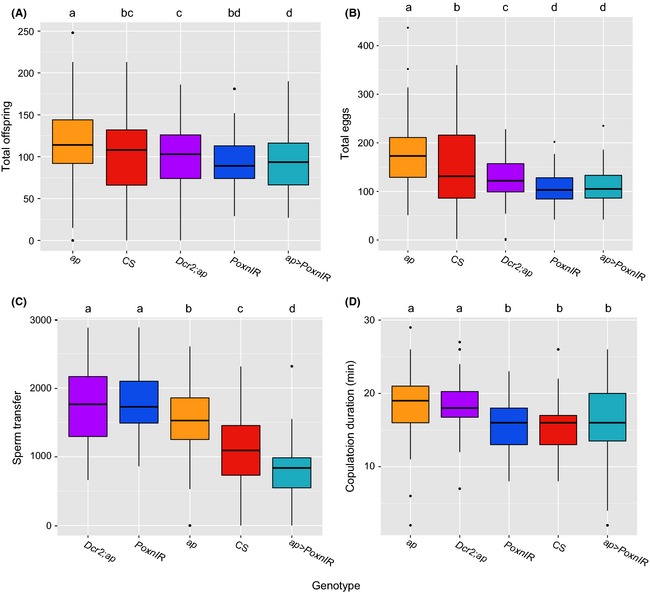
Male genotypes differ in reproductive measures and female response to posterior lobe morphology. (A) Total number of offspring, (B) total number of eggs, (C) number of sperm transferred from a single mating, (D) copulation duration. Boxplots show interquartile range (IQR), black horizontal line shows the median of each sample, whiskers show either the extreme values or ±1.5*IQR, and black dots represent outlier values. Homogeneous groups were assigned based on a statistical cutoff of *α* = 0.05. Genotype abbreviations are as follows: *ap *= *apGAL4*; CS = Canton S; *Dcr2;ap *= *UAS‐Dcr‐2; apGAL4*;* PoxnIR *= *UAS‐PoxnIR*;* ap>PoxnIR *= *apGAL4; UAS‐PoxnIR*.

Posterior lobe morphology differs significantly among the five genotypes we studied (MANOVA; *F*_12,577_ = 31.2, *P *<* *0.001). Most noticeably, intermediate‐lobed *ap>PoxnIR* males possess smaller posterior lobes (lower PC1 values) and larger L:W (lower PC2 values) on average compared to the control genotypes (Fig. 2). These males also often appear to lack the prominent “hook” structure that is characteristic of *D. melanogaster* posterior lobes. The five genotypes also show significant variation in each of the reproductive phenotypes we measured (Fig. 3). Among these differences, intermediate‐lobed *ap>PoxnIR* males tend to possess deficits in each reproductive measure compared to Canton S and the transgene controls. However, we also observed significant variation in some of the measures among the control genotypes themselves. In particular, *PoxnIR* males appear to exhibit reproductive measures that are similar in magnitude to those of *ap>PoxnIR*, which suggests the possibility that the *UAS‐PoxnIR* transgene insertion itself might have an effect on these phenotypes. Canton S males also transferred fewer sperm during mating compared to the transgene controls.

Because the four reproductive measures we obtained occur in a temporal sequence, we examined the association between successive pairs of measures to determine potential correlations among them. As expected, there is a strong correlation between the number of eggs females oviposited and the total number of offspring they produced (*r *=* *0.83). Consistent with this result, the deficit of offspring produced by females mated with *ap>PoxnIR* and *PoxnIR* males is not a consequence of reduced offspring viability, as egg‐to‐adult viability for these two genotypes was high (>0.95 survivability). Also as expected (Manier et al. 2013a), we found no strong association between copulation duration and sperm transfer amounts (*r *=* *0.16). The lower amounts of sperm transfer by *ap>PoxnIR* and Canton S males also do not appear to be a consequence of lower sperm abundance or motility, as there are no significant differences in these measures among genotypes (*χ* ^2^ = 18.5, df = 12, *P *=* *0.10; Table S1). We were unable to directly assess the association between sperm transfer amount and oviposition, as these measures were collected from two separate matings involving the same male, and we have no measure of sperm transfer from the second mating. Although oviposition rates in insects tend to be influenced primarily by seminal fluid protein composition (reviewed in Avila et al. 2011), the presence of sperm in the female storage organs does contribute to oviposition in *D. melanogaster* (Heifetz et al. 2001). This contribution is relatively small compared to that from the seminal fluid proteins, and it is unknown whether variation in sperm abundance within the storage organs affects oviposition rates. However, because the number of sperm transferred from a single mating for each male genotype we tested far exceeded that which can be stored, it seems unlikely that variation in the number of sperm transferred to the female alone would substantially affect the range in oviposition we observe. Taken together, these results suggest that variation in posterior lobe morphology might be the cause of the observed variation in reproductive measures.

We tested the effects of variation in posterior lobe morphology on pre‐ and postcopulatory reproductive measures using MANOVA with copulation duration, sperm transfer, total number of eggs laid, and total number of offspring as response variables, and PC1, PC2, and PC3 as explanatory variables. Because the number of eggs laid by a female is highly correlated with the number of offspring she produces, we constructed two separate models that included either egg number (Model I) or offspring (Model II) with the other two response variables. Male body size is known to affect copulation and fertilization success in some insects (Andersson 1994; Choe and Crespi 1997); thus, we also included tibia length as an explanatory variable in each model, as this measure provides a good approximation of overall body size in *Drosophila* males (Catchpole 1994). Tibia length showed no significant effect on pre‐ and postcopulatory reproductive measures (Model I: *F*_3,185_ = 1.36, *P *=* *0.25; Model II: *F*_3,185_ = 1.33, *P *=* *0.27). The inclusion of tibia length also did not improve the fit of the MANOVA models, and was therefore removed from the models in subsequent analyses.

We found that PC1 had a significant effect on sperm transfer, oviposition, and number of offspring and that PC2 and PC3 had a significant effect on copulation duration (Table 1). Although PC1 largely represents variation in posterior lobe area, it also includes aspects of posterior lobe shape that could contribute to this large effect. To better understand the more recognizable aspects of morphology that affect reproductive measures, we performed a similar set of analyses using posterior lobe area, L:W, and length as explanatory variables (Table S2). The results of these tests were consistent with the previous one and show that posterior lobe area was significant for its effect on sperm transfer and oviposition, although it was not significant for its effect on the number of offspring. However, whereas PC2 had no significant effect on sperm transfer in either Model I or Model II, the results from this set of tests show that L:W had significant effects on both copulation duration and sperm transfer. These tests also reveal that posterior lobe length may be the aspect of morphology that has the greatest functional importance for enabling males to achieve genital coupling and copulate: males with longer posterior lobes could achieve copulation and tended to remain in copula longer (Table 1, Fig. 1). The most obvious effects of morphological variation on postcopulatory reproductive measures can be seen when comparing intermediate‐lobed *ap>PoxnIR* and Canton S (Fig. 2). Males of both genotypes possess posterior lobes with relatively lower PC2 values and transferred fewer sperm compared to the other genotypes, but Canton S males possess posterior lobes with larger PC1 values, which correlates with greater oviposition by their mates (Fig. 3).

**Table 1:** The effect of posterior lobe morphology on pre‐ and postcopulatory reproductive measures

Principle component	Copulation duration	Sperm transfer	Eggs laid (Model I)	Total offspring (Model II)
PC1	3.14 (0.078)	**5.24 (0.023)**	**17.37 (<0.001)**	**5.64 (0.019)**
PC2	**4.09 (0.045)**	3.32 (0.070)	0.93 (0.33)	0.02 (0.896)
PC3	**7.79 (0.006)**	1.87 (0.174)	0.18 (0.67)	1.55 (0.215)

The test statistic shown in each cell is the *F* approximation of Wilk's lambda with numerator df = 1 and denominator df = 193. Test statistics significant at *α* = 0.05 are shown in bold type. *P*‐values are shown in parentheses.

### Reduced posterior lobes cause fitness losses under competitive fertilization

The single‐pair mating experiments show that in the absence of competition, males with smaller, narrower, and less hook‐like posterior lobe morphologies transfer fewer sperm during mating, and their mates lay fewer eggs. This suggests that these males might suffer a substantial fitness disadvantage in a competitive setting. To test the potential fitness costs experienced by intermediate‐lobed *ap>PoxnIR* males under competitive fertilization conditions, we performed no‐choice mating assays. In one set of crosses, we mated single *w* females with a single male from a focal genotype before mating these females with a male from a reference genotype. In a second set, we performed the reciprocal crosses by mating single females with reference males, followed by focal males. We chose three genotypes to test as focal males for these experiments: *ap>PoxnIR*, Canton S, and *PoxnIR*. We chose to include *PoxnIR* males in these experiments because even though they possess wild‐type posterior lobe morphology, they did show some reproductive deficits that were similar in magnitude to those of *ap>PonxIR* in the single mating experiments (Fig. 3).

**Figure 4:**
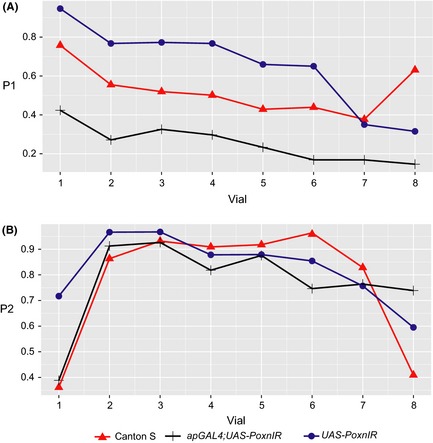
Reduced posterior lobe morphology decreases reproductive success in competitive fertilization assays. (A) Proportion of progeny sired by the focal male genotype in competitive fertilization assays against males from a reference strain when the focal male mates first (P_1_). Canton S, *n* = 15; *apGAL4; UAS‐PoxnIR, n* = 11; *UAS‐PoxnIR*,* n* = 11. (B) Proportion of progeny sired when the focal male mates second (P_2_). Canton S, *n* = 11; *apGAL4; UAS‐PoxnIR, n* = 44; *UAS‐PoxnIR*,* n* = 33.

We found significant differences in the proportion of progeny sired by focal males when focal males mated first (*F*_2,34_ = 13.5, *P *<* *0.001). When focal males mated with virgin females, the intermediate‐lobed *ap>PoxnIR* males exhibited a significantly lower P_1_ compared to both Canton S and *PoxnIR* (mean *ap>PoxnIR* P_1_ = 0.28; *P *=* *0.002; Fig. 4; Fig. S2A). Canton S males exhibited a lower P_1_ compared to *PoxnIR* males, but the difference was not statistically significant (mean P_1_: 0.62 vs. 0.72; *P *=* *0.20). The deficit in P_1_ observed for *ap>PoxnIR* is consistent with lower sperm transfer during mating, as sperm number can affect sperm competition outcomes (Manier et al. 2010; Lüpold et al. 2012). However, when comparing the average number of sperm transferred during mating for Canton S and *ap>PoxnIR* (Fig. 3C), it appears that intermediate‐lobed *ap>PoxnIR* males were disproportionately affected when they mate before a competitor male (Fig. 4, Fig. S2A). In contrast, although there were significant differences in P_2_ among the three focal male genotypes (*F*_2,85_ = 16.4, *P *<* *0.001), *ap>PoxnIR* males exhibited a higher P_2_ than Canton S (0.81 vs. 0.69), and lower P_2_ than *PoxnIR* (0.81 vs. 0.90; Fig. 4; Fig. S2A). These P_2_ results are generally consistent with the outcome expected from second male sperm precedence in insects (Simmons 2001).

Interestingly, however, females that mated with either Canton S or *ap>PoxnIR* males produced significantly fewer total offspring compared to females that mated with *PoxnIR* males, regardless of male mating order (focal male first, *F*_2,34_ = 20.8, *P *<* *0.001; focal male second, *F*_2,85_ = 34.3, *P *<* *0.001; Fig. S2B). When focal males mate first, females that mated with either Canton S or *ap>PoxnIR* males produced roughly half the number of offspring as females that mated with *PoxnIR* males at 72 h after the second mating (mean ± 1 SEM: *PoxnIR *= 43 ± 5.0, Canton S = 21 ± 3.0, *ap>PoxnIR *= 17 ± 2.0; *F*_2,34_ = 14.5, *P *<* *0.001). When focal males mated second, *ap>PoxnIR* males sired significantly fewer offspring compared to both Canton S and *PoxnIR* at 72 h after the second mating (*PoxnIR* = 31 ± 2.0, Canton S = 29 ± 3.0, *ap>PoxnIR* = 19 ± 1.0; *F*_2,85_ = 14.0, *P *<* *0.001), although the number of offspring produced by females mated with Canton S males decreased rapidly thereafter. This result was unexpected, as females that mated with Canton S males produced similar numbers of offspring in single matings compared with the other control genotypes (Fig. 3). The results of our morphometric analyses show that Canton S males do possess slightly more negative PC2 values compared to the other controls (Fig. 2), and it may be possible that this morphological difference in combination with lower sperm transfer amounts gives rise to lower offspring totals under competitive fertilization. Nonetheless, intermediate‐lobed *ap>PoxnIR* males – and the females with which they mated – suffered a substantial fitness loss in these competitive fertilization experiments compared to the other genotypes we tested.

## Discussion

Our results show that sexual selection on both pre‐ and postcopulatory functions of a single genital structure can act together to affect the rapid evolution of complex morphologies. In particular, it appears that the evolution of posterior lobe morphology in *D. melanogaster* has been driven by a composite phenomenon that includes selection on precopulatory function to secure genital coupling and postcopulatory sperm competition outcomes via sperm transfer amount. We also found that females who mate with males that possess smaller and narrower posterior lobes laid fewer eggs and consequently produced fewer offspring. The posterior lobes come into direct contact with membranous tissues of the female abdomen during copulation (Kamimura 2010), which creates an opportunity for variation in lobe morphology to influence female reproductive output as a consequence of this stimulus. Posterior lobes that are smaller and/or less structurally complex (e.g., lacking a characteristic “beak”) also cause increased female wounding at the insertion site in some *D. melanogaster* complex species (Masly and Kamimura 2014), which suggests the possibility that reduced oviposition may be a cost that females suffer as a consequence of wounding. These results are consistent with a history of cryptic female choice and/or sexual conflict, and support the idea that postcopulatory selection can in fact be a strong contributor to the evolution of complex posterior lobe morphology.

With respect to copulation duration and sperm transfer, the effects of posterior lobe morphology are likely mechanical in nature, although the male genitalia are innervated (Taylor 1989; Billeter and Goodwin 2004), which presents the possibility that males might adjust ejaculate volume or composition based on sensory feedback (see Lüpold et al. 2011; Manier et al. 2013a). The deficit in sperm transfer we observed could also be a result of female ejection of sperm from the reproductive tract (Snook and Hosken 2004), although for within‐species crosses this appears uncommon within an hour after copulation ends (Manier et al. 2010, 2013a,b). However, in crosses between *D. mauritiana* and *D. simulans*, females have been observed to eject sperm almost immediately after mating (Manier et al. 2013b). Sperm ejection in this case is attributed to species‐specific differences in ejaculate composition, but considering that these species also possess dramatically different posterior lobe morphologies, male genital morphology might also contribute to this behavior. It thus seems possible that the “fit” of the posterior lobes within the intersegmental insertion sites could represent an important sensory component of female postcopulatory reproductive responses, and might be mediated through sensory neurons that innervate the female abdominal epithelia. Careful molecular and behavioral work will be needed, however, to understand how posterior lobe morphology might direct female reproduction in this species.

The results we obtained for the effect of posterior lobe variation on some precopulatory reproductive measures in *D. melanogaster* agree with those of a similar study that used laser microsurgery to alter posterior lobe size and shape among males of the *D. melanogaster* complex species (LeVasseur‐Viens et al. 2015). Most notably, both studies show that the posterior lobe is functionally important for successful mating, and males that lack a minimal posterior lobe cannot secure genital coupling with the female. However, our results in *D. melanogaster* also differ somewhat from those that were obtained in *D. simulans,* particularly for the consequences of morphological variation on postcopulatory reproductive measures. One possible explanation for these differences is that selection pressures on the posterior lobe are different in each of these species. Another possibility is that reproductive success was quantified differently in each study. For example, we measured fertilization success by scoring the number of eggs that were laid and the number of offspring that were produced by females, whereas fertilization success in *D. simulans* was scored as a binary trait based on the presence of larvae. Both studies found no substantial differences in fertilization success when altered males mated second in competitive fertilization assays, however, when we allowed males to mate first, we found that males with altered posterior lobe morphologies suffer significantly reduced reproductive success compared to controls.

Although we used genetic manipulations to alter posterior lobe morphology, it is unlikely that the genotypes we constructed gave rise to abnormal phenotypes other than those of the posterior lobe that significantly influenced reproductive success. First, we outcrossed each of the transgenes we used to Canton S, thus minimizing behavioral or seminal fluid protein differences that might affect copulation duration or oviposition. Although we cannot completely exclude complex epistatic interactions that might affect reproductive behaviors or differences in seminal fluid protein composition or amount as the cause of variation in postcopulatory reproductive success, we expect that all genotypes would have been equally affected, particularly if seminal fluid differences explain our results. Second, we restricted RNAi knockdown of *Poxn* to the cells in the developing genitalia that give rise to the posterior lobe. Although both *Poxn* and the *apGAL4* transgene are expressed in cells that give rise to somatic reproductive tissues, they are not expressed in the germ line or in the accessory glands. Moreover, neither gene appears to be expressed in the adult male internal reproductive organs that are necessary for sperm transfer during copulation (*e.g*., the ejaculatory bulb).

*Drosophila melanogaster* males that possess larger posterior lobes with prominent hook‐like morphologies experience greater reproductive success than males with smaller and less hook‐like lobes. These phenotypes are generally consistent with the results from experimental evolution studies that show elevated sexual selection can give rise to larger and/or more structurally complex genitalia (Simmons et al. 2009; House et al. 2013; Simmons and Firman 2014). Our results identify both pre‐ and postcopulatory mechanisms of sexual selection as contributing to the evolution of posterior lobe morphology. Our results also lend some support the idea that sexual selection on genital morphology could contribute to the evolution of reproductive isolation between populations, similar to what is observed from sexual selection on other traits (Phelan and Baker 1987; Boughman 2001; Svedin et al. 2008; Head et al. 2013; Manier et al. 2013b; Schwander et al. 2013; Seddon et al. 2013; Wojcieszek and Simmons 2013; Castillo and Moyle 2014; Dyer et al. 2014; Hudson and Price 2014; Latour et al. 2014). Although several studies have shown that divergent genitalia do not appear to be a common cause of strong reproductive isolation among species (Shapiro and Porter 1989; Coyne and Orr 2004), genitalia may indeed prove to be an important contributor to the speciation process via reinforcement (Howard and Gregory 1993; Questiau 1999; Servedio and Noor 2003; Hoskin and Higgie 2010). Isolated populations within species often display substantial variation in the morphology of male genital structures. Upon secondary contact, the fitness losses that occur as a consequence of variation in genital morphology could help increase selection for mate discrimination between these populations. Although the present data do not specifically address this idea, the role of genital evolution in reinforcement is an interesting possibility that warrants further investigation (McPeek et al. 2011; Masly 2012; Simmons 2014).

## Conflict of Interest

None declared.

## Supporting information


**Movie S1.** Courtship and copulation behavior of a Canton S male mated with a w female. Codecs: H.264, Linear PCM.

**Movie S2.** Courtship and copulation behavior of a *Dcr2; ap>PoxnIR; elavGAL80* male mated with a *w* female. Codecs: H.264, Linear PCM.

**Table S1.** Male sperm motility and abundance.
**Table S2.** The effect of posterior lobe morphology on pre‐ and post‐copulatory reproductive measures.
**Figure S1.** Measures of courtship behavior among males with reduced posterior lobe morphologies.
**Figure S2.** Males with reduced posterior lobe morphologies suffer fitness losses compared to normal males.
