# Supplementary material for: Multiple sexual selection pressures drive the rapid evolution of complex morphology in a male secondary genital structure
Source: Ecol Evol. 2015 Sep 23;5(19):4437–50. doi: 10.1002/ece3.1721 (PMC4667835; doi:10.1002/ece3.1721)
Supplement: Supplementary file 3 — Table S1. Male sperm motility and abundance. Table S2. The effect of posterior lobe morphology on pre‐ and post‐copulatory reproductive measures. Figure S1. Measures of courtship behavior among males with reduced posterior lobe morphologies. Figure S2. Males with reduced posterior lobe morphologies suffer fitness losses compared to normal males. [file ECE3-5-4437-s003.docx]

**Supporting Information**

**Male sperm abundance and motility assay**

We measured the abundance and motility of sperm in the testes of males from each of the genotypes we tested in our experiments using the assay described in Orr (1992) and Masly et al. (2006). Virgin males were aged for three to four days in isolation before their testes were removed, dissected in 1X PBS, and immediately examined under a compound microscope with dark-field optics at 100X magnification. We classified each male into three sperm motility classes: “Many” for males with abundant motile sperm present that fill the field of view; “Few” for males that show a few localized motile sperm present; “None” for males that possess no motile sperm. Males were scored blind with respect to their genotype. We tested the following number of males for each genotype:

*UAS-PoxnIR*, n=80

*w*^1118^, n=151

*UAS-Dcr-2; apGAL4*, n=74

*apGAL4; UAS-PoxnIR*, n=72

Canton S, n=116

*UAS-Dcr-2; apGAL4; UAS-PoxnIR*, n=154

| **Table S1. Male sperm motility and abundance.** | | |  |
| --- | --- | --- | --- |
|  |  |  |  |
| **Genotype** | **Many** | **Few** | **None** |
|  |  |  |  |
|  |  |  |  |
| *PoxnIR* | 68 | 10 | 2 |
| *w118* | 131 | 18 | 2 |
| *Dcr2;apGAL4* | 61 | 12 | 1 |
| *apGAL4>PoxnIR* | 67 | 3 | 2 |
| Canton S | 100 | 13 | 3 |
| *Pin / CyO* | 102 | 2 | 1 |
| *Dcr2;apGal4>PoxnIR* | 140 | 12 | 2 |
|  |  |  |  |

| Table S2. The effect of posterior lobe morphology on pre- and post-copulatory reproductive measures. | | | | |
| --- | --- | --- | --- | --- |
| **Principle component** | **Copulation duration** | **Sperm transfer** | **Eggs laid (Model I)** | **Total offspring (Model II)** |
|  |  |  |  |  |
|  |  |  |  |  |
|  |  |  |  |  |
| Area | 0.42 (0.520) | **5.74 (0.018)** | **13.2 (<0.001)** | 3.05 (0.082) |
| L:W | **6.05 (0.015)** | **11.9 (<0.001)** | 0.28 (0.595) | 0.26 (0.610) |
| Length | 1.89 (0.171) | **4.08 (0.045)** | 0.03 (0.870) | 0.002 (0.965) |
|  |  |  |  |  |
| The test statistic shown in each cell is the *F* approximation of Wilk's lambda with numerator df=1 and denominator df=193. Test statistics significant at α=0.05 are shown in bold type. *P*-values are shown in parentheses. | | | | |
|  |  |  |  |  |

**
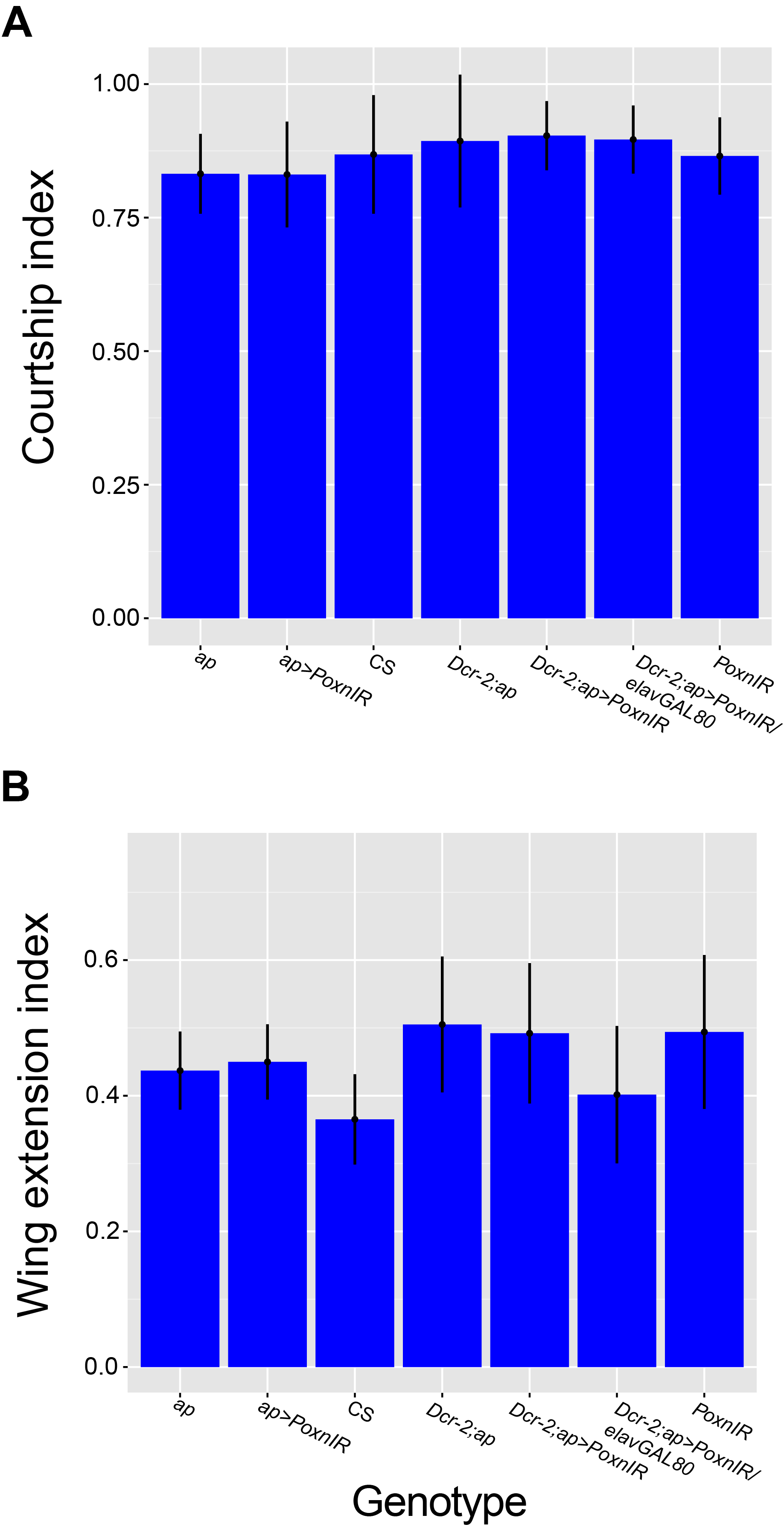
**

**Figure S1.** Measures of courtship behavior among males with reduced posterior lobe morphologies. **(A)** Courtship index; **(B)** Wing extension index. Error bars show ±1 SEM.


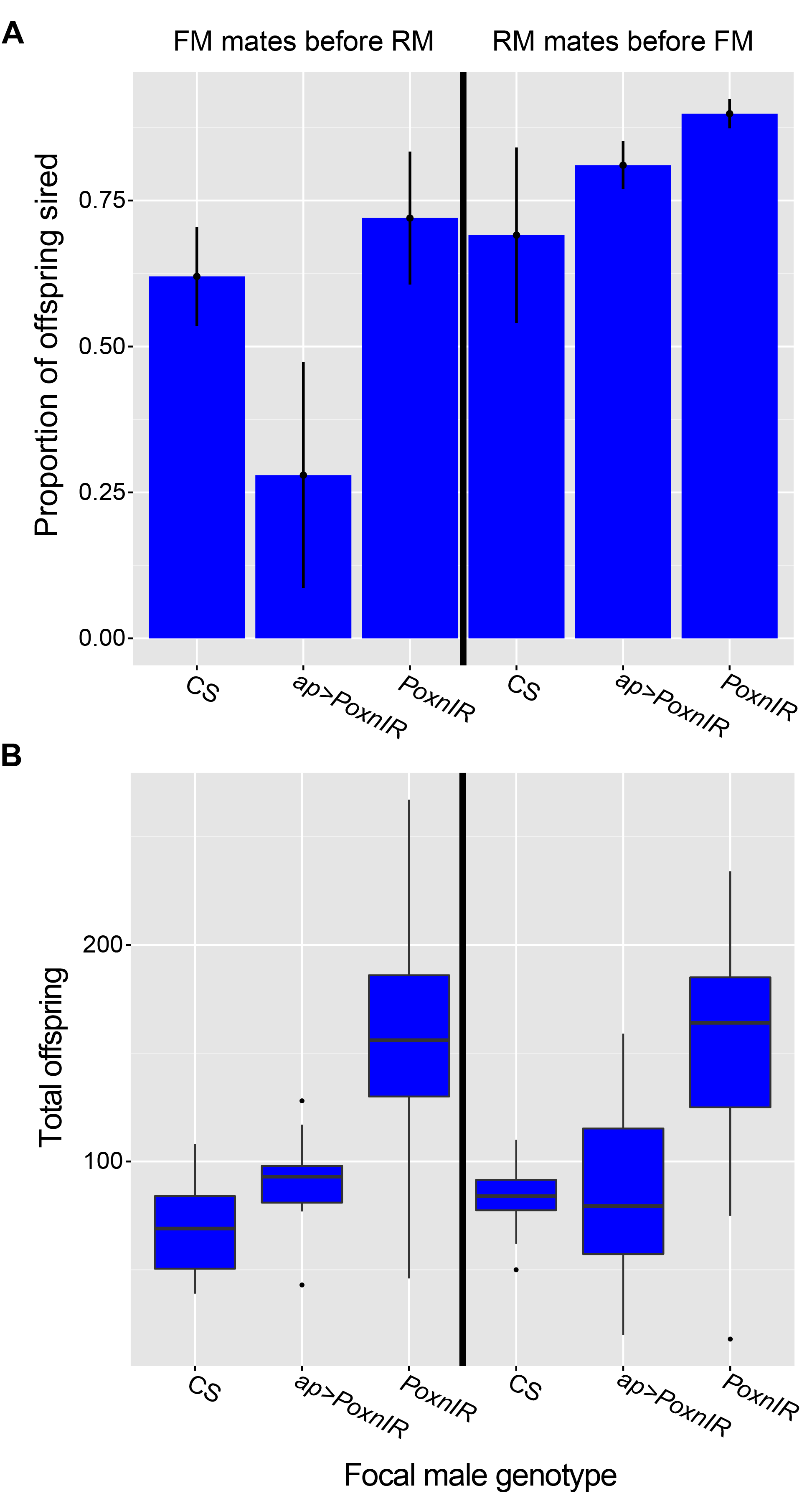


**Figure S2. Males with reduced posterior lobe morphologies suffer fitness losses compared to normal males.** (A) Proportion of total offspring sired by the focal males (FM). Error bars show ±1 SEM. (B) Total number of offspring produced by females mated with FM and reference males (RM). Boxplots show interquartile range (IQR), black horizontal line shows the median of each sample, whiskers show extreme values or ±1.5*IQR, black dots represent outlier values. The left side of panels A and B show the focal male followed by reference male crosses and the right sides of each panel shows the reciprocal cross. Genotype abbreviations are: CS=Canton S; *PoxnIR*=*UAS-PoxnIR*; *ap>PoxnIR*=*apGAL4; UAS-PoxnIR*.

**Movie S1.** Courtship and copulation behavior of a Canton S male mated with a *w* female. Codecs: H.264, Linear PCM.

**Movie S2.** Courtship and copulation behavior of a *Dcr2; ap>PoxnIR; elavGAL80* male mated with a *w* female. Codecs: H.264, Linear PCM.

**Literature Cited**

Masly, J. P., C. D. Jones, M. A. Noor, J. Locke, and H. A. Orr. 2006. Gene transposition as a cause of hybrid sterility in *Drosophila*. Science 313:1448-1450.

Orr, H. A. 1992. Mapping and characterization of a "speciation gene" in *Drosophila*. Genet. Res. 59:73-80.
